# The concept of Messiah in abrahamic religions: A focused study of the eschatology of Sunni islam

**DOI:** 10.1016/j.heliyon.2022.e09080

**Published:** 2022-03-09

**Authors:** Qais Salem Alma'itah, Zia ul Haq

**Affiliations:** College of Shariah & Islamic Studies, University of Sharjah, United Arab Emirates

**Keywords:** Messiah, *Sunni*, *Mahdi*, Judaism, Christianity, Islam, Eschatology

## Abstract

The purpose of this research is to conduct a comparative analysis of the Messiah in Semitic religious discourse, focusing on Muslims' specially the Sunni school of thought. The notion of Mahdism is generally recognized in all three major sematic faiths. Muslims scholars, particularly Shiites, believe in Mahdi and see him as the cornerstone of their faith, to the point where Shiites theology is difficult to fathom without it. We argued in this article that the Shiites' concept of Mahdism is derived from both Jewish theological literature, which is based on the concept of a savior who will arrive at the end of time, and Christian theology, which considers Jesus as a Redeemer who came for the salvation of Humanity after grave sin. Sunnis, who constitute the majority of Muslims, hold a different view of Messiah. The objective of this study was to establish the actual Sunni Muslim position on the issue of Mahdism and salvation, as obtained from authentic sources of Islam.

## 1 Introduction

The concept of Messiah is one of the most divisive components of all three Abrahamic religions, and it is one of the most disputed among them. The three divine faiths all believe in the existence of a figure who will appear at the end of time and serve as a reinforcer of their various religious beliefs (Haqqy, 2020, p. 87). Every time and place throughout history when and where mankind has been subjected to political, social, and religious persecution, the hope for a messiah or redeemer to rescue people from their position has become bigger and more pervasive. Despite the fact that Islam's founding texts remain silent on the issue of a Mahdi (Messiah), there is a rising debate among Muslims regarding whether or not there is a savior who can save Muslims in these difficult times. This is due to a number of different factors. For example, there is widespread dissatisfaction with Muslim economies' inability to improve living standards, as well as with social, economic, and intellectual injustice in third-world countries, among other things. In this article, we hope to gain a better understanding of the concept of Mahdism in Semitic discourse through a cross-disciplinary comparison of religious texts. We will pay particular attention to determining the actual Islamic notion on the subject as expressed in primary Islamic chronicles, as well as demonstrating the implications of this belief on Jews, Christians, and Muslims.

The concept of Messiah was not explicitly stated in the Holy *Qur'an* or any authentic sayings of the Holy Prophet (SAW). Certain elements were incorporated from paganism. Everything in Islamic literature about the concept of Messiah is based on misunderstanding and fallacies.

This study aims to address numerous critical questions, the most important of which are as follows:

1. Does the concept of Messiah have pagan roots, and if so, to what extent were people who believed in it influenced by paganism?
2. How do Jews perceive Mahdism and how does it affect them ideologically and politically?
3. How do Christians view the Messiah, and what effect does this have on them?
4. What are Shiites and Sunnis' views on Mahdism?

The study is organized into three chapters, each of which contains two sub chapters. The study begins with a preface that details the approach, method, and literature review. The first chapter provided an in-depth introduction to the concepts of Mahdism and salivation, as well as the concept's theological underpinnings. The second chapter examines the concept of Messiah as it is described in Jewish and Christian literature, emphasizing on the concept's ramifications for both. The final chapter explores the notion of Mahdism in Islam, taking into account both *Sunni* and *Shia* perspectives on the concept and its ramifications for their religious and political ideologies, before concluding with a discussion of the study's significant findings and recombination's.

## 2 Literature review and theoretical framework

There is a considerable amount of attention has been paid in Semitic discourse, either supporting or some time opposing the concept of Mahdism. However, the majority of these studies were undertaken from a proponent Christian standpoint, which considers it to be one of the fundamentals of Christian theology, and the subject has not attracted opponents who do not believe in that type of theology thus far. It's worth noting that each Abrahamic religion believes in a single God and expects the Messiah to emerge as predicted in their scriptures: Christians look forward to Jesus Christ's second coming, whereas Jews look forward to the Messiah, David's son... Muslims, on the other hand, expect the coming of two people at the same time: The Messiah, Jesus son of Mary, and the predicted Mahdi. Muslims, according to *Ibn al-Qayyim,* are waiting for the Messiah, Jesus, son of Mary, to come down from heaven and the Mahdi, who would bring justice to the earth (Ibn Qayyim, 1999, p. 286).

In Islamic perspective, the concept of Mahdism is not mentioned in any of the major Islamic holy texts. Although the holy Qur'an mentions Jesus and his mother Marry in various places and accords them high prestige, it does not mention Jesus' rebirth. In the same vein, the books of authentic narrations compiled by *Imam Bukhrî* and *Imam Muslim* did not contain any information concerning Jesus' rebirth. Even though there is no reference to the Mahdi in Abu Hurairah's famous hadith which mentions the ten signs of the Final Hour. As a result, the majority of Muslims do not consider Mahdism to be a real concept. A few of Hadith compilers, such as Ibn Maja and *Abu D'aud*, did, however, mention certain narrations from the Prophet Muhammad (SAW) regarding the possibility of Jesus' rebirth.

Many of Mahdism's opponents quote *Ibn Khaldun's* writings. He is referred to be as the "Father of Social science" who considers the Mahdism a toxic Shiites ideology that has infiltrated Sunnism through Sufism (Ibn Khaldun, p. 486) While some contemporary *Sunni* Muslim scholars appear to endorse the Mahdi concept. There are many Sunni scholars giving significance to the narratives about Mahdi due to their trust to the sources of hadith, even the others named hadiths about Mahdi as isolated (*a'ahad*) and not sufficient to prove any fundamental Islamic doctrine.

This brief review of Islamic literature illustrates that, despite the absence of reliable proofs from the Qur'an and Hadith, Mahdism has existed for nearly as long as Islam and shows no signs of lessening in the near future. As a result, it has become essential to include relevant Islamic literature into academic discourse in a comparative manner. This study is being conducted to address this proposal.

## 3 Research methodology

An inductive analytical approach is applied to track the opinions and views of people of various religions in Mahdism, as well as the sayings of scholars and their opinions on this belief, followed by a detailed comparison to determine areas of agreement and disagreement, and finally, these opinions are addressed analytically to determine the implications of the said belief in Mahdism.

## 4 Definition of mahdism and similar terms

Messianism or Mahdism is the belief in the advent of a messiah who acts as the Saviour of a group of people. Messianism originated as an Abrahamic religious belief, but other religions have messianism-related concepts. Religions with a messiah concept include Judaism (*Mashiach*), Christianity (Christ), Islam (*Isa Masih*), Zoroastrianism (*Saoshyant*), Buddhism (*Maitreya*), Hinduism (*Kalki*) and Taoism (Li Hong). In Judaism, the messiah will be a future Jewish king from the line of David and redeemer of the Jewish people and humanity(Ginzburg, 2001, p. 18). In Christianity, Jesus is the Messiah, the Saviour and redeemer. In Islam, Jesus was a prophet and the Messiah of the Jewish people who will return in the end times.

The Arabic term *Mahdi* (مهدي) literary refers to a guided person (*Ibn Manzur,* p. 27). In Islamic eschatology, a messianic deliverer who will fill earth with justice and equity, restore true religion, and usher in a short golden age lasting seven, eight, or nine years before the end of the world.

The term refers to the process of escaping an intolerable or unpleasant environment or condition. It is a vital and crucial topic in Christianity since it believes that man is saved from his sins through the process of redemption carried out by Christ on the cross (*al-Zah'ar*, 2012, p. 209).

According to *Abd al-Ghani Salama*, the Saviour, Messiah, or *Mahdi* are all titles for the prophet who has been awaited by believers for a long time. However, each sect awaits him for a different cause, which is generally to deliver them from their misery and pain, as well as to fill the globe with justice and mercy, a desire shared by all. This is a more objective definition because the author does not appear to be biased or have predetermined thoughts about his beliefs.

## 5 The origin of mahdism

It is common for most man-made religions to include a concept of the Messiah or the Awaited Mahdi, which is often accompanied by various tales and mysteries. Many people believe that the belief in the Awaited Mahdi found in the majority of religions implies that the concept is natural and inevitable. However, according to the Holy Qur'an, a majority's agreement on a wrong thing does not imply that it is reliable. (And if you listen to the majority on earth, they will direct you away from God's path) [*Al-An'am:* 116].

According to some scholars, religious believers who were subjected to religious persecution were more likely to come to believe in a Messiah. As time progressed, the belief in a Messiah became more widespread and was associated with a multitude of mythology around across religions.

Messiah is the only source from which all oppressed groups drew the features of their doctrinal heritage in the struggle of good and evil, light and darkness, truth and falsehood... and this is what is embodied for us by many texts of all sects, whether Islamic or Christian and Jewish religions, and even ancient doctrines and beliefs, which required a hero whenever they faced difficulties and injustice in their societies.

According to Muhammad Siddiqi: "A careful examination of the anthropology of ancient beliefs reveals that similar images have been woven for the personality of that devoted Messiah, and the Mahdi (Messiah) has become the only reliable source for all oppressed groups in their struggle against evil, darkness, and falsehood. The concept is demonstrated by the major faiths, whether Islamic, Christian, or Jewish, as well as ancient sects and beliefs, which required a hero in the face of hostility and persecution and thus created a picture dominated by the mythical character of their Messiah, woven by the imagination of those persecuted in every human group, not just Muslims" (*Siddiqui,* 2013, p. 18).

People are driven to seek rescue signified by an ideal capable of altering reality, transforming evils into pure good for their supporters while unleashing chaos and disaster on their adversaries, according to Siddiqi's explanation of the phenomenon. He also points out that the doctrine (of the expected *Mahdi*) is a contentious topic in Islamic theology, with significant dispute over the nature of this Messiah who will return at the end of time to "fill the earth with justice as it was filled with injustice," among other things. The personas of this savior were treated differently by the various Islamic sects. By the passage of time, the conflict became deeper, and a large number of sects arose around the concept of the Messiah. A messiah who would aid them and deliver them from evil was envisioned differently by each group, which was a source of contention (*Siddiqui,* 2013).

*Faleh Mahdi* brought together numerous Islamic, Jewish, Christian, and pagan concepts about (the Awaited Messiah), their philosophical roots and foundations in ancient civilizations, as well as the political context in which the concept of (Awaited Messiah) emerged. This Messiah's personality changed according to man-made beliefs; for example, to the ancient Egyptians, he was the Nile; to the ancient Iraqis, he was the god *Tammuz*; to Hindus, he was the personality of "*Krishna*" and "Ram"; and to Buddhists, he was lord Buddha (Falih, 1981, p. 6).

Beginning with the ancient Mesopotamians and continuing through ancient Egyptians and Persians, we can trace this concept all the way back to the awaited Messiah among the Jews and Christians, where we can find characteristics of the Savior Christ among Christians, the Savior of the Redeemer among the Jews, and finally to the Mahdi thought in Islam and the political conflicts between Muslims that manifested in their clearest forms between the Sunnis and Shite and resulted in the idea of a special Mahdi for each them (Falih, 1981).

As previously stated, we do not need to present additional examples or evidence to establish that the Messiah notion has its roots in ancient religions because the evidences are overwhelming and no one denies them. Some believers in the Messiah, on the other hand, believe that the presence of the Messiah in pagan faiths serves to support its validly and the necessity of the Messiah for human nature.

This examples establish the fact that the Messiah notion has its roots in ancient religions even some believe that the presence of the Messiah in pagan faiths serves to support the validity of the concept of a Messiah and its consistency with human nature.

## 6 The concept of messiah in judaism and its implications

Messiah, according to "*Tanakh*," is a prophet, a sign from the Lord for Jews, and his name is "Emmanuel" (Matthew 1/21, 23). and who will appear on the Lord's Dreadful Day. (Malachi 4:5) He is the sprout that grows from Jesse's stump, as well as the sapling that bears fruit. (Isaiah 11:1.) He is the one who will reign from Jacop's descendants. (Luke 1/33.) He is the sovereign who will sit on David's throne. (Isaiah 9:7) He is a Melchizedek priest for all eternity. He is the one who will raise the scepter from "*Judah*," and he will not allow the scepter to pass through his feet. He is the one who will sit by the Lord's right until He makes his enemies his footstool. (Mezmurlar 110/1.) He is the one who will rule as a king, make wise decisions, and bring justice. *Judah* will be saved and Israel will be safe during his time. He will be known as "*Jehovah*, our righteous savior." In the prophet Daniel's dream, his kingdom is described as follows: "In my vision at night, I looked, and there before me was one like a son of man, coming with the clouds of heaven." He was led into the presence of the Ancient of Days as he approached him. He was bestowed with authority, glory, and sovereign power, and all nations and peoples of all languages worshiped him. His dominion is an everlasting dominion that will never perish, and his kingdom will never be destroyed. (Daniel 7:13–14) Messiah will perform various miracles, such as healing blinds, lame people, and deaf people. Furthermore, he is the one who will ride into Jerusalem on a donkey and be greeted with cheers. Aside from that, other nations will follow his light after he has enlightened the Jews. (Isaiah 60/1–3) Messiah, who possesses extraordinary abilities, is also described as a servant who suffers for Israel. His worth will not be recognized by his people, but they will repent later due to their ignorance of Messiah. (Isaiah 53:11) Messiah is also told in Ezekiel's words, and these messages are given to Jews: just as Ezekiel resurrects dead bones and leads them with God's permission, Messiah will resurrect Jews from their state of death and bring them back to life. He will gather the sons of Israel from all over the world, forming them into a single nation and a steady staff in God's hand. He is the one who will bring them together so that they do not become separated, and he will become their sole king and shepherd. He will make certain that they strictly follow God's laws, and he will make the lands they settled theirs for all time.

The Jewish sects disagree on the question of Messiah, between those who are zealous in their conviction like Hasidim sect to the point of creating a special room in their homes for the expected Messiah and rebuking God each night for making it too late to resurrect the promised Messiah. The Messiah, whom the Jews believe will be a descendant of David, and whose arrival will put an end to all their problems, and they have numerous tales about his imminent arrival, to the point where some of them used to say to his family if he desired to sleep: (If the Messi'ah came while I was sleeping, wake me up without hesitation) (p. 141).

While some Jews, such as reformers, reject the concept of Messiah and do not believe in Him at all, the vast majority of Jews are constantly on the lookout for the arrival of the "anticipated Messiah." The title "Messiah" comes from the Hebrew word "Messiah," which literally translates as "one who has been anointed with blessed oil." When it comes to "messianic" it is a term that alludes to the concept of salvation. When describing Israeli discourses, the term messianic is commonly used to distinguish between discourses that are logical, modern, and pragmatic and discourses that are illogical, fanatical, and radical (*Salam'ah,* 2019).

The Jews (the Maccabees) were patiently waiting for the Messiah to arrive during the Roman period of control over Palestine. A number of individuals claiming to be the Messiah arrived around the same time. Jesu s of Nazareth, called the Messiah, was one of them (Yılmaz, 2018).

When "Jesus" arrived in the shape of a saint rather than a king, to liberate the Jews from the persecution of the Romans, many rejected him and incited the Roman ruler to punish him.

After Christ, Christianity and Judaism split off from one other. Judaism, however, is still awaiting their messiah, who in their belief will arrive as a king of the lineage of David, who will free them from slavery and scattering.

In Judaism, there is a minority of followers who believe that the Messiah will arrive once tyranny, wars, and oppression are widespread on Earth and that He will free people of their sins and eradicate all religions except Judaism, after which He will fight a brutal war between good and evil to defeat the Christian and Muslim followers. The war known as the end of history, or Armageddon. This subjugates the Jewish people to a position of dominance over the rest of the world. From that time on, the Lord of lords will sit, and there will be a period of peace (p. 224).

According to this view, the resurrection of Messiah represents the reawakening of Judah's kingdom, and heaven is intended for the enjoyment of witnessing events in which the Jews triumph over all the peoples of the earth, as they were responsible for the scattering of the children of Israel and their persecution.

The Jews, according to another Jewish sect, are not the superior of all human beings, nor are they the best of all humanity. Instead, for extended periods of time in history, they were not in the same position as other nations; they were tormented and humiliated as a penalty for their sins. From here, they shifted their attention to the hunt for a Messiah who would rescue them from their humiliation and elevate them to a more honorable human status. They referred to him as the "messenger of God" on earth (Al Hanafi, 1980).

This concept had a negative impact on Jewish philosophy for the following reasons:

1. Waiting for Messiah with a vengeful tyrant who triumphs over his followers and exacts revenge on their enemies undoubtedly has an effect on the personality of the people awaiting such a Messiah, the most notable of which is intolerance toward other groups, and it is from this that we learn that some sects offer human sacrifices until the Messiah arrives (Hamza, 1980). In this context *Haqqy* maintains that this waiting for the Messiah had a profound influence on the religious and political aspects of the Jewish nation (*Haqqy,* 2020, p. 87).
2. The search for a Messiah took many forms and groupings among Jews. One school of thinking ties the concept of Messiah to the difficult conditions faced by the Jewish people during and after the Babylonian captivity, which resulted in their persecution, while another school of thought believes that waiting for Messiah is not appropriate in Jewish faith. Rather, it is drawn from other religions, such as the Persian religion, in which many of their judgments and actions were conditional on the coming of the Awaited Messiah, causing many of their rites and rituals to be disrupted.
4. Religions such as Christianity and Judaism both believe in the coming of the Awaited Messiah, but Christians believe that this expectation was fulfilled once by the Messiah, Jesus, son of Mary, who will appear for a second time. Christians believe that Christ is (the Messiah) of the people and that he is the one who will put Judaism back on the right path. This Messiah was referred to as (Jesus Christ) or the Son of God by the Jews. Many prophecies in the Book of the New Testament, which speak of the Messiah of the end of times, make reference to him.
5. It has retained a prominent position in contemporary American thought, owing to the Protestant belief that Old Testament served as a prelude to the New Testament and that the Torah is the Bible's mother. Americans believe that the establishment of a Jewish state in Palestine, must come first before the arrival of the Awaited Messiah. Accordingly, the United States supports the establishment of a Jewish state with Jerusalem as its capital, as well as the protection and assistance of Jews in this endeavor.

## 7 The christian concept of messiah

Christian understanding of Messiah is grounded in the notion of redemption and salvation from the great sin. Unlike Judaism, which awaits the Messiah's return to fulfill his last role in helping the Jewish people, we consider the Christian messiah as having already fulfilled his function in redeeming creation and his own body on the cross (Hussain, 2017).

Because he is the most important part of the trinity creed, Jesus Christ is the centralized person around whom the New Testament is formed, and Jesus Christ is the one who comes to save his people from their sins. (Matthew 3/21/2019) This salvation is extended not only to his people, but to everyone who believes in him. (Rom. 3:21–24) He is God's incarnation for this purpose. (Colossians 1:14–15, 17, 18) In other words, he is God's son who comes to be offered as redemption for humanity's original sins, as well as for God's justice and the goodness of those who believe in Jesus. (Rom. 3:25–2673) Such an offering reveals a transformation of a fundamental concept in "Tanakh": Imposing the redemption of Jewish mistakes on Messiah transformed into an original sin that people carried since their birth, and Messiah is transformed into an offering of sacrifice who pays the price as a result of God's justice. Joseph A. et al. (Joseph A. et al., 1990) As a result, this conceptual evolution is the most important indicator of the separation of Judaism and Christianity, with the latter transforming into a different religion. According to Matthew, Jews expect Jesus, who introduces himself as the Messiah, to demonstrate some signs in accordance with the concept developed in "*Tanakh*" (Matthew 27/39–44). However, considering Christianity's redemption doctrine, this is not possible because it is necessary for Jesus to die on the cross in order for God's justice to be done, and thus the destiny to save his believers from their sins is fulfilled. As a result, even though Jesus is deeply troubled by the burden of such responsibility (Matthew 22/46), he must carry it out by praying for those who are unaware of it. On the other hand, in Christianity, Messianic thought is separated from the context formed by Judaism in relation to this world in terms of religious-political basis and presented within the framework of a completely celestial kingdom. (Luke 23:33–34).

However, when it comes to Christ's return, the church generally adheres to Bible literalism which considers Christ's return as one of the unseen events whose date is unknown except to God. The Bible makes around 300 references to Christ's return, and it says in the Gospel of "Matthew 24":"What sign do you have of your coming and the end of the age?" "Be cautious!" Jesus forewarned them. Do not be fooled; many will claim to be the Christ in my name! And they're going to fool a lot of people, and you're going to hear about wars and war rumors. Do not be concerned, do not be afraid. It has to be every single one of them, but the game is not yet over (Rehman, 2020).

This concept has unquestionably had an impact on Christian personality and thought because Christians see Jesus as one of the essentials to their belief system. Despite this, Christians are not predisposed to violence against others because they do not wait for a Messiah who will triumph over the other world (Sammak, 1993, p.13).

## 8 The muslims view of messiah

Although the Qur'an contains numerous references to monotheism and rejection of polytheism, as well as numerous verses discussing the Day of Judgment, there is no verse in the *Qur'an* that mentions either the Messiah or the Mahdi, and no unauthentic prophetic narration makes any reference to either of this concept. This created a massive schism between Sunni belief in the Mahdi, which is consistent with the Holy *Qur'an*, and Shiite belief, which has elevated Mahdism to the status of Islam's cornerstone and quoted dozens of hadiths from the imams in support of their position. However, in his book Contemporary Muslim Apocalyptic Literature (2005), David Cook, a professor of religious studies at Rice University in Houston, argues that Islam most likely began as an apocalyptic movement, and it has maintained a strong apocalyptic and messianic character throughout its history, a character that has manifested itself in literature as well as in periodic social explosions (Cook, 2008,g p. 24).

## 9 The Shiites concept of messiah

Among the Twelver Shiites, the predicted *Mahdi* is *Muhammad ibn al-Hasan al-Askari,* the last of their imams, who is said to have been born in Samarra in the year 255 AH. Shiites refer to him as the Mahdi, or owner of the time. They give him a great reverence and pray for his appearance whenever his name is mentioned saying: "*Ajjal allahu farjahu*" which means: "God hasten his reappearance," whenever his name is mentioned (Zaheer, 1995, p. 251).

Certain religious rulings that were largely performed in the presence of the Imam have become null and void in the absence of the Imam. For centuries, Shiite scholars prohibited jihad, Friday prayer, and the establishment of a state, until the concept of a jurist's representative (*Wilayat al Faqih*) in legal matters on behalf of the hidden imam was invented, and the idea of establishing a state floated during the Safavid era at 1500 AD.

Many Shi'a scholars feel that the concept of Messiah is one of the most fundamental components of Islam, and they argue that it is nonsensical to deny it because it is embraced by all major religions around the world, including Christianity (*Al-Mahdi*, 1429 AH).

Additionally, the Shiites believe that the presence of the Messiah in all religions indicates that this belief is not alien to human natural disposition or reason, given that the majority of human beings believe in the existence of a Messiah who will rescue humanity from its miserable reality and transport it to another dreamed reality and transport it to another dreamed reality (*Mustafa,* 2018).

While many theorists attribute it to persecution shared by adherents of other religions, as we discussed in our study of the Messiah's pagan origins," according to *Muhammad Siddiqui,* "any anthropologist of ancient religions will discover that an identical imagery has been woven into the Messiah's identity." It is a human resource from which all oppressed groups derived the characteristics of their doctrinal heritage in the struggle between good and evil, light and darkness, truth and falsehood, and why the concept is embodied in numerous texts by all religions, whether Islamic, Christian, or Jewish, as well as ancient doctrines and beliefs that required a Messiah when narrowed down. As a result, they created an image dominated by a mythological figure, weaved together by the imaginations of persecuted people of all faiths (Siddiqui, 2013).

The Shiites were divided on the identity of the expected Mahdi. Several of their groups, like as the Sabaeans believe that *Ali ibn Abi Talib* will return and bring justice to the planet. And the Twelvers stated that he is *Muhammad ibn al-Hasan al-Askari.* In the following we will also discuss the discrepancies between Shiites toward *Muhammad ibn al-Hasan al-Askari* in terms of his appearance date, location, and description (p. 243).

The Twelver Shi'ites used a variety of evidence attributed to *Ahl al-Bayt* imams to try to argue that *Muhammad ibn al-Hasan al-Askari* is the expected Mahdi, including the hadith "The Hour will not come until a man from my family follows my name." According to their order, *Muhammad ibn al-Hasan al-Askari* came after his father, the eleventh imam, bringing the total number of imams to twelve. They also claimed that he lived a short period among his father's colleagues, known as the Minor occultation, before disappearing from view and entering his major occultation, which lasts till the end of time (Al-Tusi, 1997, p. 235).

Some researchers have pointed out that the rest of the non-Imami Shiite sects disagree in this view of their twelfth imam, and they accuse *Imamiyyah* jurists of formulating this doctrine, especially since *al-Qummi* - states in his book *al-Maqalat wa al-Firaq:* "He *(Al-Hasan Al-Askari)* died, and no successor was seen for him, and no son was known to him, thus they disagreed about his successor,". He composed this book in the second part of the third century AH, at the start of what the Imamis call the Minor Occultation. Above all the Shiites' disintegration following the death of their eleventh imam, as *Al-Ash'ari* describes in his classic work *"al-maqalat*": "At that time (in the year 260), *Abu Muhammad* was jailed, and his Shiites and allies dispersed" (Al Qummi, 1982, p.102). *Shaheed Sayyid Muhammad Sadiq al-Sadr,* one of the most important *Imami* thinkers of the modern period, opposes all of these facts and insists on the notion of Imam Mahdi and its place among the *Imami* Shiites.

Despite Shiites' devotion to *Mahdi,* their language surrounding him was confusing, leading many academics to regard him as a fictitious figure who exists only in the thoughts of Shiites who believe he is Mahdi and await his emergence from his great occultation. Several Shiite discrepancies include the following:

1. Shiites argue about the identity of Muhammad ibn Hasan's mother and whether he was born or not?! Shiite scholars disagree about his birth, with some asserting that *Al-Hasan Al-Askari* died and left no son (Al *Ghaw'aji,* 2001, p. 401).

Further, they assumed that when *al-Askari* died, his brother *Ja'far* acquired his fortune, which Ja'far would not have received if he had a son in accordance with Qur'anic inheritance law.

Others sought to authenticate Muhammad's birth, including *Nawbakhti,* who said it occurred on Friday in the middle of the month of *Sha'ban* (255) AH, according to the most renowned sayings, and *Kulayni* asserts in (Al Kafi), that he was born in 256 H (Kulaini, 2006 AD).

Moreover, people who confirmed his birth disagreed on the precise day and location of his birth. According to some of them, he was born eight months after his father, *Al-Hassan*, died. Others stated that he was born five years before his father died (Nobakhti, 2012, p. 115).

2. They also disagreed on the year he disappeared. Some of them place it in the year 256 AH, some in the year 258 AH, and still others in the year 255 AH (p. 235).
3. The Shiites disagreed on the location of their *Mahdi, Muhammad ibn al-Hasan al-Askari.* According to some, he disappeared at Samarra, in the basement of his father's house, and this is one of the most famous Shiite sayings, widely circulated among them and included in their writings. Others believe he disappeared in *Makkah Al-Mukarramah,* while others claim he was in Yemen's *Shamroukh* valley, while according to the others, he was in Al–Ta'if (Zaheer, 1995).
4. They also disagreed about the return, when and how it will occur (*Alusi,* 2001, p. 201).

The study of the notion of Messiah among Shiites reveals that it is an idea that has crystallized specifically within Shiite Islam after being acquired from ancient belief systems such as Jewish-Christian or Iranian-Zoroastrian. This includes the belief in *Ghaiba,* which is the absence of the imam whose life is miraculously prolonged, and the expectation of his return, an anticipated return of the Mahdi from death or *Ghaiba* at the end of time to liberate his followers from persecution (*Akdid*, 2017).

## 10 The sunni perspective on the messiah

The Mahdi, according to Sunni theology, is an ordinary person, but he is supported by God just like any of His righteous guardians, and most importantly, the Sunnis do not await his advent because their decrees and laws are complete from the moment the Prophet died. "Today, I have finished My favor on you, and I have sanctioned the reign of Islam for you," God said. They see his appearance as one of the signs of the final day of judgment, when he will appear to establish the state of justice and Islamic law. He does not appear to be a dictatorial ruler or avenging angel. He, like everyone else, is a begotten and born human being. Most importantly, because some prophetic traditions mention the concept of *Mahdi,* Sunnis do not disparage *Mahdi* followers. Sunni's views on the concept of Messiah are summarized as follows:

"The awaited *Mahdi* is true, and it will occur at the end of time, near the emergence of the (*Dajja'l)* Antichrist, and before the arrival of Jesus, when there is a disagreement when a caliph dies, so the Mahdi emerges and pledges allegiance, and justice is established among the people for seven years or more, and in his time Jesus Ibn Maryam, peace and blessings be upon him, descended upon the earth,". The long-awaited Mahdi, who is portrayed in reliable hadiths as descended from the Prophet Muhammad's lineage. He will bring justice to the people, promote the law, and free them from injustice (Baz, 1434 AH bin Baz, 1995).

The majority of *Sunni* scholars think that the Mahdi's matter is established via several prophetic narrations and that he will arrive at the end of time, but without reference to myths and legends (*Al-Abbad**,* 1400).

The second point of view is that of Sunni intellectuals who oppose the Mahdi, as articulated by Ibn *Khaldun* in his *Muqaddima'h* (Khuldun*,* 1936, p. 555).

And Sheikh Mahmoud Shaltout, the previous Sheikh of *Al-Azhar*, rejected not only the Mahdi's hadiths, but all hadiths concerning the Messiah. Other important thinkers who challenged the Mahdi doctrine include Muhammad Rashid Rida, Muhammad Farid Wajdi *Ahmed Amin*, and *Abdullah bin Zaid Al Mahmoud* (*Wajdi*,1980, p. 481).

The Mahdism, according to some Sunni scholars, is not a fundamental belief because there is no mention of him in the Holy Qur'an, and it is impossible for the Holy Qur'an to ignore such a crucial issue although it has been mentioned in a number of dubious prophetic narratives that are not legitimate. This is not an idea that can be found in the Qur'an. The Messiah concept has persisted in popular mythology since ancient times, passing through ancient religions to the present day, where we can find echoes of these concepts in the Abrahamic religions: Judaism, Christianity, and Islam, as well as the religions f the Indian subcontinent (Sirnini, 2011).

Furthermore, the Mahdi's hadiths were not narrated in Al-Bukhari or Muslim, the two most trustworthy sources for prophetic accounts. When Sunni scholars discovered that several of the hadiths cited in the Mahdi are insufficient or invented, they concluded that belief in the Messiah is not a core tenet of Islam.

Some of them inclined to disregard the Mahdi's hadiths for a pragmatic reason when they saw numerous persons throughout history have claimed to be Messiahs, all of which is based on a belief that is not addressed in the Qur'an and is supported by hadiths, the finest of which are solitary. Thus, in order to shut the door on phony Mehdis, these scholars concluded that no Mahdi has to come at all.

The above explanation demonstrates that the *Sunni* Messiah is distinct from the Shiite Messiah in a number of respects, including his name, father's name, genealogy, age, qualities, and expected actions.

## 11 Conclusion

The concept of the Messiah is deeply embedded in the majority of religions, and several myths and superstitions have developed in various religious traditions surrounding the Messiah's nature. Certain sects of believers in the Awaited Messiah have argued about the concept's originality, arguing that it is either consistent or inconsistent with human nature. Despite the fact that not all Jewish sects agreed on it, the concept of a Messiah has spread widely among Jews as a result of social, political, and religious factors passed down through history; thus, the concept of a Messiah has become one of the cornerstones of Jewish faith. The concept of the Messiah, according to the study, is not a fundamental Islamic belief, and the majority of Muslim academics believe it originated in Jewish-Christian or Iranian-Zoroastrian traditions, particularly in Shiite Islam. We can conclude from this research that the Mahdi is a person supported by God, just like any of his righteous guardians, and that the Sunnis do not wait for him to arrive because their rulings and laws are complete as of the Prophet Muhammad's death, and thus anyone among the Sunnis who denies him is not considered a disbeliever, but it is necessary to believe in due to the large number of prophetic narratives surrounding it. This study recommends strengthening religious dialogue between different religions, as well as between sects affiliated with Islam, and establishing a relationship based on unity, coexistence, and dialogue in order to achieve common interests, as well as correcting the discourse of Islamic beliefs in a way that provides the correct foundations for a sound religious understanding. It also suggests that researchers and scholars focus on studies that clarify the truth and demonstrate the correct position on some beliefs that some people believe are fundamental to Islamic faith but are actually a result of a misunderstanding of the holy text.

## Declarations

### Author contribution statement

Qais Salem Alma'itah: Conceived and designed the experiments; Analyzed and interpreted the data; Contributed reagents, materials, analysis tools or data; Wrote the paper.

Zia ul Haq: Performed the experiments; Contributed reagents, materials, analysis tools or data; Wrote the paper.

### Funding statement

This research did not receive any specific grant from funding agencies in the public, commercial, or not-for-profit sectors.

### Data availability statement

Data will be made available on request.

### Declaration of interests statement

The authors declare no conflict of interest.

### Additional information

No additional information is available for this paper.
